# Does capitation payment under national health insurance affect subscribers’ trust in their primary care provider? a cross-sectional survey of insurance subscribers in Ghana

**DOI:** 10.1186/s12913-016-1622-0

**Published:** 2016-08-24

**Authors:** Francis-Xavier Andoh-Adjei, Dennis Cornelissen, Felix Ankomah Asante, Ernst Spaan, Koos van der Velden

**Affiliations:** 1 NHIA 36-6th Avenue, Ridge. PMB Ministries Post Office, Accra, Ghana; 2 GROW-School of Oncology and Developmental Biology, Department of Clinical Epidemiology and Medical Technology Assessment (KEMTA), Maastricht University Medical Centre-The Netherlands, Maastricht, Netherlands; 3 Institute of Statistical, Social and Economic Research (ISSER) University of Ghana, Legon-Accra, Ghana; 4 Radboud Institute for Health Science, Department for Health Evidence, Radboud University Medical Center-Netherlands, Nijmegen, Netherlands; 5 Radboud Institute for Health Science, Department for Primary and Community Health, Radboud University Medical Centre-Netherlands, Nijmegen, Netherlands

**Keywords:** Capitation payment, Subscriber trust, Primary care provider, National health insurance, Ghana

## Abstract

**Background:**

Ghana introduced capitation payment for primary care in 2012 with the view to containing escalating claims expenditure. This shift in provider payment method raised issues about its potential impact on patient-provider trust relationship and insured-patients’ trust in the Ghana National Health Insurance Scheme. This paper presents findings of a study that explored insured-patients’ perception about, and attitude towards capitation payment in Ghana; and determined whether capitation payment affect insured-patients’ trust in their preferred primary care provider and the National Health Insurance Scheme in general.

**Methods:**

We adopted a survey design for the study. We administered closed-ended questionnaires to collect data from insurance card-bearing members aged 18 years and above. We performed both descriptive statistics to determine proportions of observations relating to the variables of interest and chi-square test statistics to determine differences within gender and setting.

**Results:**

Sixty-nine per cent (69 %) out of 344 of respondents selected hospital level of care as their primary care provider. The two most important motivations for the choice of a provider were proximity in terms of geographical access (40 %) and perceived quality of care (38 %). Eighty-eight per cent (88 %) rated their trust in their provider as (very) high. Eighty-two per cent (82 %) actively selected their providers. Eighty-eight per cent (88 %) had no intention to switch provider. A majority (91 %) would renew their membership when it expires. Female respondents (91 %; *n* = 281) were more likely to renew their membership than males (87 %; *n* = 63). Notwithstanding capitation payment experience, 81 % of respondents would recommend to their peers to enrol with the NHIS with rural dwellers (87 %; *n* = 156) being more likely to do so than urban dwellers (76 %; *n* = 188). Almost all respondents (92 %) rated the NHIS as (very) good.

**Conclusion:**

Health Insurance subscribers in Ghana have high trust in their primary care provider giving them quality care under capitation payment despite their negative attitude towards capitation payment. They are guided by proximity and quality of care considerations in their choice of provider. The NHIA would, however, have to address itself to the negative perceptions about the capitation payment policy.

**Electronic supplementary material:**

The online version of this article (doi:10.1186/s12913-016-1622-0) contains supplementary material, which is available to authorized users.

## Background

As policy makers focus attention towards achieving health policy goals of quality, efficiency and access, they are faced with increasing cost of health care partly due to inefficiencies in the financing arrangements [1]. While reforms in provider payment systems may help address such inefficiencies, it could also affect quality of care and patients trust in health care providers. This paper presents findings of a study that sought to explore insured-patients’ perception about, and attitude towards capitation payment in Ghana; and to determine whether capitation payment affect their trust in their preferred primary care providers (PPP) and the National Health Insurance Scheme (NHIS) in general.

Trust is defined as “the willingness of a party to be vulnerable to the actions of another party based on the expectation that the other will perform a particular action important to the truster, irrespective of the ability to monitor or control the other party” Mayer et al. (1995); In Hall et al. [2]. Thom et al. [3] describe patients’ trust as “necessary for an effective health care system”, adding that the presence or absence of trust in patient-provider relationships in health care delivery can influence the provision and use of health care services. Various theories and measurement scales on patients’ trust in care delivery have been developed over the years to measure patient’s trust in their primary care physicians. These include the Trust in Physician Scale [4], the Primary Care Assessment Survey [5] and the Patient Trust Scale [6]. Hall et al. [2], on the basis of existing theoretical models and other works, conceptualized patient trust as having potentially 5 domains: fidelity, competence, honesty, confidentiality and global trust. Their model distinguishes itself from existing models by describing the association between physician trust and other constructs. They posit that physician trust is related to insurer trust in that patients who trust their care providers may worry less about their insurer; that trust is related to, but distinct from satisfaction; and that provider trust is related to potential determinants or outcome of trust. They identified the dimensions of trust as the patient’s general satisfaction with health care, satisfaction with the physician, length of time with the provider, patient’s willingness to recommend the provider to friends and their intent to switch provider. Thom et al. [3] found trust to be a significant predictor of continuity with primary care provider, adherence to treatment recommendations and satisfaction of care. They concluded that “understanding patients’ trust (…) can lead to changes in health care delivery that protect doctor-patient relationship” and that it could help “identify physician and patient behaviours that tend to promote or block patient trust.” Patient-provider trust relationship is therefore very crucial to effective healthcare systems.

But the growing demand for health care service within budgetary constraints in low and middle income countries and the increasing cost of health care services have necessitated reforms in the provider payment methods [7–11] that may affect patient-provider relationship. Economic theory has it that changes in provider payment systems attract responses from providers[12], which could affect the quality and quantity of service they provide[13] and thereby affect patients trust in their care provider. A provider payment method that can potentially contain cost is capitation payment that is currently being piloted in Ghana under the National Health Insurance system. Evidence on potential impact of capitation on patient-provider relationships has been noted in studies conducted in high income countries [14] and the evidence is mixed: Kao et al. [14, 15] found that patients whose primary care providers were paid by capitation method were less likely to trust their providers. Other studies reported of patients accessing healthcare from capitated providers expressing discomfort with them being paid by capitation method [16], and were more likely to switch their primary care providers [17]. Hall et al. [18], on the other hand, did not establish any negative effect of capitation payment on patients’ trust for their primary care providers.

**Fig. 1:**
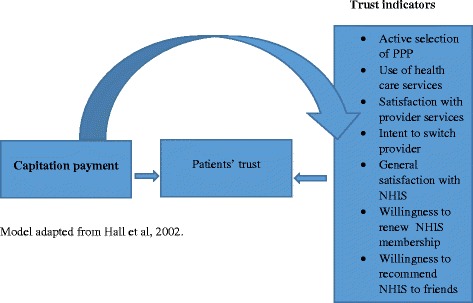
Conceptual model of effect of capitation payment on patients’ trust in primary care

In 2003, Ghana introduced a National Health Insurance Scheme (NHIS) to provide financial access to health care services. Initially, the National Health Insurance Authority (NHIA) paid its credentialed providers by Fee-For-Service (FFS) method but had to switch to Diagnosis-Related-Grouping (DRG) payment due to abuse of the system [19, 20]. Years into the implementation of DRG payment, the NHIA observed that the abuse in the system still persists. The NHIA decided to further reform its provider payment system with the introduction of capitation payment beginning with a pilot in the Ashanti region of Ghana, with all other regions continuing with the DRG payment. The main objective for the capitation payment mechanism is to contain cost while ensuring quality of service provided to insured clients. However, this policy shift raised issues about the potential impact of capitation payment on patient-provider trust relationship in Ghana. The capitation policy was introduced amidst protest from service providers, civil society organizations and politicians for its perceived negative effects on primary care delivery [21]. In a press release, the Society of Private Medical and Dental Practitioners notified the public that “*We suspend our services to NHIS subscribers indefinitely. The system is detrimental to quality of health care provision and a major threat to the survival of private health facilities*” (The Ghanaian Times newspaper dated 02/01/2012). The Pharmaceutical Society of Ghana is reported to have sued the NHIA over capitation payment on the grounds that it “*poses grave danger to patients given among other factors the recognition it (capitation payment) gives to persons outside the pharmacy profession*” (The Daily Guide newspaper of 23/01/2012). Capitation payment continues to be a public discourse in Ghana, with proponents rationalizing its importance and usefulness for health care delivery in Ghana; and opponents calling for its withdrawal. Although studies have been done on Ghana’s National Health Insurance Scheme, to our current knowledge, only few of the studies focused on the capitation payment policy. A study by Agyei-Baffour et al. [10] focused on subscriber and health worker knowledge, perceptions and expectations of the capitation payment while that of Aboagye [22] focused on determining the likelihood of the capitation payment achieving the intended benefits for Ghana. Neither of these studies covered the insured patient’s trust of providers in delivering quality care under the capitation payment. Besides, studies relating to patients and provider relationship have focused on patient satisfaction [23–27] but as noted by Hall et al. [2] although trust is related to satisfaction, the two are distinct constructs. This study could, thus, be one that focuses on patient trust in their primary care provider under capitation payment and helps fulfil the need for empirical evidence on the issue, in particular for low/middle-income countries. Understanding insured patients’ trust in their care providers will enable policy makers to implement effective and efficient strategies that will help improve the capitation implementation process and also help improve primary care delivery in Ghana. Building on existing theory, we applied the conceptual model in Fig. 1 below to our study.

We adopted Hall et al.’[2] model in identifying indicators for measuring insured-patients’ trust in primary care delivery under capitation payment. In our model, we attempt to assess insured subscribers’ trust in primary care provision as well as in the National Health Insurance Scheme under the capitation payment system. Trust in primary care provider is measured by subscribers’ active selection of PPP, their use of health care services, their satisfaction with provider services, and their intent to switch primary care provider while trust in the NHIS is measured by general satisfaction with the scheme, willingness to renew membership upon expiry and willingness to recommend NHIS to friends.

## Methods

### Study design

We adopted a survey design for the study. We earlier did a review of the literature to understand the theoretical effects of capitation payment on patients’ trust in primary care and a newspaper review of the issues that heralded the introduction of capitation payment in Ghana to inform the survey questionnaires design.

### Study setting

The study took place in the Ashanti region of Ghana. The Ashanti region was the first region where the implementation of capitation payment started in 2012. It has a population of 4,780,380 and accounts for 19.4 % of the total population of Ghana of which about 61 % is urban (Ghana Statistical Service. Population and Housing Census, 2010). There are 1,126,216 households in the region with an average household size of 4.1. As of 2013 when this study was being designed, there were 619 NHIS-accredited health facilities. The region had 24 district offices of the National Health Insurance Authority (NHIA) and 1,585,098 active card-bearing members representing about 34 % of the regional population.

### Sampling

Multi-stage sampling was applied to recruit subjects for the survey. The list of Enumeration Areas (EAs) used for the 6th round of the Ghana Living Standard Survey (GLSS6) was used as the sample frame. We adopted the sample design used by the Ghana Statistical Service (GSS) for post enumeration survey (PES) to determine the number of EAs (clusters) in the region. Each of the ten regions in Ghana was treated as domain for selection and analysis, and was based on probability sample of 250 EAs of which 45 were in Ashanti. We used the World Health Organization (WHO) revised Expanded Programme of Immunization (EPI) survey reference manual as guide for determining the sample size per cluster. Using the WHO recommended table indicating number of subjects per cluster; we opted for the desired precision level of ±3 % and expected coverage of 95 % to determine the number of subjects for the survey. We arrived at 10 subjects per cluster (EA) giving a representative sample of 450 households (subjects) to be interviewed. Having determined the number of households for the interviews, we sought assistance from the GSS to select 30 EAs from the 250 with probability proportional to size. Fifteen households (and 15 replacements) were then randomly selected from each of the 30 EAs. A list of the households and their replacements were given to the interviewers for the field interviews. Apart from its strength in facilitating call back at any household where an interviewer may encounter challenges at the initial visit, this strategy also has the advantage of discouraging the interviewers from selecting households based on their subjective judgment while in the field and thereby potentially introducing bias into the study [28]. In each household an adult card-bearing member aged 18 years and above was interviewed.

### Data collection and analysis

We administered closed-ended questionnaires to collect data on respondents’ socio-demographic characteristics, choice of PPP and rationale behind choice, their trust in the PPP, satisfaction with care delivery and the NHIS, as well as their perception about, and attitude towards capitation payment. The target population was active card bearing members of 18 years and above. Data cleaning resulted in the removal of respondents whose health insurance ID card number was not indicated to authenticate their insurance membership. This brought the total number of respondents included in the initial descriptive statistical analysis to 383. With the use of SPSS (v.20.0.0.1) software we performed descriptive statistics on the socio-demographic characteristics of respondents based on the 383 respondents. Thereafter, we excluded 39 respondents who indicated they had no knowledge of capitation and had no PPP from the analysis of trust, perception and attitude. We performed descriptive statistics of respondents’ choice of provider and rationale behind their choice based on gender and setting. In determining respondents’ trust in their primary care provider and in the NHIS, we performed both descriptive statistics to determine proportions based on gender and setting relating to the variables of interest and chi-square test statistics to determine differences in observations within gender and setting.

## Results

### Socio-demographic characteristics of respondents

Three hundred and eighty-three (383) out of 450 sampled subjects participated in the survey and this represent 85 % response rate (Table 1). About 53 % were resident in an urban setting, 83 % were females, 23 % had no formal education and 23 % were unemployed. A majority of respondents (65 %) had been active members of the NHIS for two years or more at the time of the survey. Almost all respondents (94 %) had used their NHIS card to access health services in the two years prior to the survey.

**Table 1:** Socio-demographic characteristics of respondents

Variables (N=383)	Frequency (%)	Urban (%)	Rural (%)
Gender			
Male	66 (17)	40 (60)	26 (40)
Female	317 (83)	162 (51)	155 (49)
Age			
18-31	121 (32)	61 (50)	60 (50)
32-45	119 (31)	65 (55)	54 (45)
46-59	66 (17)	31 (47)	35 (53)
60-74	42 (11)	28 (67)	14 (33)
75+	35 (9)	17 (49)	18 (51)
Marital status			
Married	230 (60)	119 (52)	111 (48)
Never married	61 (16)	34 (56)	27 (44)
Divorced	20 (5)	13 (65)	7 (35)
Widowed	60 (16)	29 (48)	31 (52)
Other	12 (3)	7 (58)	5 (42)
Education			
Tertiary	27 (7)	21 (78)	6 (22)
Secondary/SHS	57 (15)	42 (74)	15 (26)
Middle/JSS	157 (41)	74 (47)	83 (53)
Primary	54 (14)	27 (50)	27 (50)
Never attended school	88 (23)	38 (43)	50 (57)
Employment status			
Formal	50 (13)	28 (56)	22 (44)
Self-employed	209 (55)	98 (47)	111 (53)
Retired	34 (9)	22 (65)	12 (35)
Unemployed	90 (23)	54 (60)	36 (40)
Length of NHIS membership			
≤2 years	30 (8)	16 (53)	14 (47)
2-3 year	24 (6)	9 (38)	15 (62)
3-5 years	57 (15)	25 (44)	32 (56)
> 5 years	167 (44)	88 (53)	79 (47)
No response	105 (27)	64 (61)	41 (39)
Used NHIS card past 2 years			
Yes	360 (94)	193 (54)	167 (46)
No	23 (6)	9 (39)	14 (61)

Source: Authors’ household survey, 2014

### Choice of preferred primary care provider

Table 2 presents details about the choice of provider and rationale behind choice. Sixty-nine per cent (69 %) out of 344 of respondents selected hospital level of care as their PPP. The two most important motivations for the choice of a PPP were proximity in terms of geographical access (40 %) and perceived quality of care (38 %).

**Table 2:** Subscriber preferred choice of provider and rationale behind choice

Parameter	All (%)	Gender		Setting	
		Male (%)	Female (%)	Urban (%)	Rural (%)
	N=344	n=63	n=281	n=188	n=156
Level of care					
Hospital level	238 (69)	51 (81)	187 (66)	137 (73)	101 (65)
Clinic level	51 (15)	4 (6)	47 (17)	21 (11)	30 (19)
Lower levels	12 (4)	3 (5)	9 (3)	5 (3)	7 (5)
Not indicated	43 (12)	5 (8)	38 (14)	25 (13)	18 (11)
Reason behind choice					
Proximity	139 (40)	25 (40)	114 (41)	137 (73)	101 (65)
Perceived quality	129 (38)	25 (40)	104 (37)	21 (11)	30 (19)
Staff attitude	27 (8)	7 (11)	20 (7)	5 (3)	7 (5)
Indifferent	49 (14)	6 (9)	43 (15)	25 (13)	18 (11)

Source: Authors’ household survey, 2014

### Subscriber trust in primary care provider under capitation payment

We asked the 344 respondents to rate their trust in their PPP as well as in the NHIS bearing in mind their experiences with the health care system under the capitation payment (Table 3). Eighty-eight per cent (88 %) of them rated their trust in their primary care provider as (very) high. There was no difference in the level of trust between male and female respondents [χ^2^ = 2.010; *p* = 0.570] nor between urban and rural dwellers [χ^2^ = 3.799; *p* = 0.150]. Eighty-two per cent (82 %) actively selected their primary care providers. Eighty-eight per cent (88 %) had no intention to switch PPP. There was no significant difference in intention to switch provider between male and female [χ^2^ = 3.799; *p* = 0.150]; nor between urban and rural settings [χ^2^ = 2.839; *p* = 0.242]. However, the minority that intend to switch provider would do so for perceived poor service quality, non-availability of skilled staff, and perceived poor medication. With regard to respondents’ trust in the NHIS, the study found that a majority (91 %) would renew their membership when it expires. Female respondents (91 %; *n* = 281) were more likely to renew their membership than males (87 %; *n* = 63) though the difference was not significant [χ^2^ = 1.486; *p* = 0.476]. Notwithstanding capitation payment experience, 81 % of respondents would recommend to their peers to enrol with the NHIS with rural dwellers (87 %; *n* = 156) being more likely to do so than urban dwellers (76 %; *n* = 188); [χ^2^ = 6.874; *p* = 0.009]. Almost all respondents (92 %) rated the NHIS as (very) good. However, only about 22 % of respondents agreed to rate capitation payment.

**Table 3:** Subscriber trust of primary care provider

Variable (N=344)	All (%)	Male (%)	Female (%)	Chi; *p*-value	Urban (%)	Rural (%)	Chi; *p*-value
*Trust in Provider*	N=344	n=63	n=281		n=188	n=156	
Actively selected provider							
Yes	281 (82)	57 (90)	224 (80)	4.543; 0.103	154 (82)	127 (81)	3.790; 0.580
No	63 (18)	6 (10)	57 (20)		34 (18)	29 (19)	
Rate trust of provider							
(Very) high	304 (88)	55 (87)	249 (87)	2.010; 0.570	157 (84)	147 (94)	3.799; 0.150
Low	38 (11)	7 (11)	31(11)		29 (15)	9 (6)	
Null	2 (1)	1 (1)	1 (2)		2 (1)	0	
Intention to switch provider							
Yes	41 (12)	3 (5)	37 (14)	3.799; 0.150	26 (14)	14 (9)	2.839; 0.242
No	303 (88)	60 (95)	243 (86)		161 (86)	142 (91)	
*Trust in NHIS*							
Would renew card upon expiry							
Yes	312 (91)	55 (87)	257(91)	1.486; 0.476	165 (88)	147 (94)	4.553; 0.103
No	32 (9)	8 (13)	23 (9)		22 (12)	9 (6)	
Would recommend NHIS to peer							
Yes	279 (81)	47(75)	232 (82)	2.127; 0.145	143 (76)	136 (87)	6.874; 0.009
No	65 (19)	16 (25)	49 (18)		45 (24)	20 (13)	
Rate satisfaction with NHIS							
(very) high	315 (92)	60 (95)	255 (91)	2.890; 0.576	170 (90)	145 (93)	2.891; 0.576
(very) low	29 (8)	3 (5)	26 (9)		18 (10)	10 (7)	
Null		0	1		0	1	
Rate capitation payment							
(very) good	75 (22)	10 (16)	65 (23)	1.711; 0.425	38 (20)	37 (24)	2.891; 0.576
Null	269 (78)	53 (84)	216 (77)		150 (80)	119 (76)	

Source: Authors’ household survey, 2014

### Subscriber perception about the positive attributes of capitation payment

Exploration of respondents’ perception about the positive attributes of capitation payment as contained in the information, education and communication (IEC) messages of the NHIA revealed that between 69 % and 81 % (strongly) agree with the statements that captured the positive attributes of capitation payment (Table 4). There were only two attributes on which respondents differed significantly in opinion. Among respondents who (strongly) dis-agreed with some of the positive attributes of capitation payment, residents in urban areas (26 %; *n* = 188) expressed significantly higher dis-agreement on the notion that capitation encourages better diagnosis and treatment than residents in rural areas (9 %; *n* = 156) [*p* = 0.000] and on the notion that capitation makes treatment easy and effective (25 % and 13 %, respectively) [*p* = 0.035].

**Table 4:** Subscriber perception about the positive attributes of capitation payment

Statement category (N=344)	Answer categories			
	(Strongly) agree	(Strongly) dis-agree	Don't Know	^χ^; *P*-value
	Frequency (%)			
Capitation will drive down cost	239 (69)	75 (22)	30 (9)	
Male (n=63)	37 (59)	18 (29)	8 (12)	4.687; 0.196
Female (n=281)	202 (72)	57 (20)	22 (8)	
Urban (n=188)	126 (67)	47 (25)	15 (8)	3.645; 0.302
Rural (n=156)	113 (72)	28 (18)	15 (10)	
Capitation will improve quality of care	203 (59)	118 (34)	23 (7)	
Male	29 (46)	27 (43)	7 (11)	7.190; 0.066
Female	174 (62)	91 (32)	16 (5)	
Urban	110 (59)	68 (36)	10 (5)	4.583; 0.205
Rural	93 (60)	50 (32)	13 (8)	
Capitation stops provider shopping	248 (72)	51 (15)	45 (13)	
Male	37 (58)	15 (24)	11 (18)	7.602; 0.055
Female	211 (75)	36 (13)	34 (12)	
Urban	131 (70)	28 (15)	29 (15)	2.077; 0.557
Rural	117 (75)	23 (15)	16 (10)	
Capitation encourages better diagnosis & treatment	278 (81)	63 (18)	3 (1)	
Male	48 (76)	14 (22)	1 (2)	1.919; 0.589
Female	230 (82)	49 (17)	2 (1)	
Urban	138 (73)	49 (26)	1 (1)	17.935; 0.000
Rural	140 (90)	14 (9)	2 (1)	
Capitation makes treatment easy and effective	273 (79)	67 (19)	4 (2)	
Male	47 (75)	15 (24)	1 (1)	2.171; 0.538
Female	226 (80)	52 (19)	3 (1)	
Urban	139 (74)	47 (25)	2 (1)	8.636; 0.035
Rural	134 (86)	20 (13)	2 (1)	

Source: Authors’ household survey, 2014

### Subscriber attitude towards capitation payment

Notwithstanding the high trust in their PPP and in the NHIS under the capitation payment ; and their positive perception about the attributes of capitation, respondents in general, exhibited negative attitude towards capitation payment (Table 5). About 58 % have a feeling that implementation of capitation payment in the Ashanti region was politically motivated while 65 % perceive capitation payment as causing frustration to the insured patients at the health facilities with another 49 % indicating that capitation contributes to deaths in the implementing region. There were no significant differences in attitude towards capitation between male and female, nor between urban and rural dwellers.

**Table 5:** Subscriber attitude towards capitation payment

Statement category (N=344)	Answer categories			
	(Strongly) agree	(Strongly) dis-agree	Don't Know	^χ^; *P*-value
	Frequency (%)			
Capitation is meant to punish the people in Ashanti	199 (58)	80 (23)	65 (19)	
Male (n=63)	38 (60)	16 (25)	9 (15)	1.200; 0.753
Female (n=281)	161 (57)	64 (23)	56 (20)	
Urban (n=188)	112 (60)	45 (24)	31 (16)	1.811; 0.613
Rural (n=156)	87 (56)	35 (22)	34 (29)	
Capitation was brought to Ashanti because of politics	199 (58)	80 (23)	65 (19)	
Male	38 (60)	16 (25)	9 (15)	1.200; 0.753
Female	161 (57)	64 (23)	56 (20)	
Urban	112 (60)	45 (24)	31 (16)	1.811; 0.613
Rural	87 (56)	35 (22)	34 (22)	
Capitation contributes to the death of the people	169 (49)	103 (30)	72 (21)	
Male	33 (52)	15 (24)	15 (24)	2.160; 0.540
Female	138 (49)	88 (31)	57 (20)	
Urban	102 (54)	49 (26)	37 (20)	4.679; 0.197
Rural	67 (43)	54 (35)	35 (22)	
Capitation causes frustration at the health facilities	223 (65)	84 (24)	37 (11)	
Male	46 (73)	14 (22)	3 (5)	3.973; 0.264
Female	177 (63)	70 (25)	34 (15)	
Urban	131 (70)	39 (21)	18 (9)	5.053; 0.168
Rural	92 (59)	45 (29)	19 (12)	

Source: Authors’ household survey, 2014

## Discussion

### Choice of preferred primary care provider

We found in our study that health insurance subscribers are guided by proximity and quality of care considerations in their selection of preferred primary care provider under the capitation payment policy. This is consistent with findings from Yeboah et al. [29], Uchendu et al. [30], Muriithi [31] and Buor [32]. The economic and quality of care consideration that guided subscribers’ choice of provider suggests that they intuitively understand that the cost of health care services is not only the amount of money that they pay at the health facilities or the premium they pay to the insurance scheme but also the incidental and opportunity costs that come with attending facilities that are too distant from one’s place of residence and seeking care from providers who may fall short of providing quality care. It is also significant to note that urban dwellers were more critical on the effect of capitation on quality of care, a plausible reason being the level of education of the urban dwellers which is higher than that of rural dwellers and the possible exposure of the former over the latter to media discussions and agitations about the capitation payment policy.

One emerging trend that seem disturbing to the district health delivery system in Ghana under capitation payment is the threat of crowding out lower level facilities by those of district hospital level. A majority of respondents selected facilities that are of district hospital status and clinics where they perceive to have access to better qualified health personnel to manage their health conditions leaving only a few for the lower levels of care that serve as the first point of entry in the health delivery system. If the trend is not addressed the smaller facilities in rural communities may risk being thrown out of business since enrolees may want to enrol with higher level provider facilities thereby making health care more expensive. Another consequential effect would be that if the smaller facilities are not able to survive the competition and fold up, there will be lack of geographical access to residents in deprived areas where such smaller facilities may be located to serve the unmet health needs of subscribers and the general public. Lack of access to health care services may lead to lack of confidence in the NHIS and plausibly de-motivate subscribers from renewing their membership upon expiry. Potential subscribers may also not enrol since their membership cards will be meaningless to them without health facilities where they may attend for quality care in times of need.

This situation may, however, be addressed by introducing the “list patient” and “geographic” capitation payment models [8, 33] whereby the reimbursement of providers with capacity to attract enrolees is tied to the number that enrol with the facility; and that of smaller facilities tied to the insured population living within a particular geographical area. In the case of Ghana and other comparable low/middle-income countries, the “list patient” capitation model may be applied to hospitals and clinics that have the capacity to attract more enrolees while “geographic capitation” model is applied to the community-based health planning and services (CHPS) compounds with their capitated rates tied to the number of active card bearing members of the NHIS within their catchment area. As capitation is being scaled up nationwide, one way to address this emerging trend would be for policy makers to consider introducing these models into the scheme design to support the lower level facilities to remain relevant in the health delivery system. This will strengthen the gatekeeper system by encouraging the use of first level facilities for cases that may not necessarily require the specialist attention of the district hospitals.

### Subscriber trust in primary care provider

With regard to patient-provider trust relationship, our study did not establish significant negative findings on subscriber trust in their primary care providers, a finding that is consistent with those of Hall et al. [18] and Pereira et al. [16]. The high level of trust in the care provider as reflected in the level of satisfaction that respondents exhibited by rating their health providers highly and showing little intention to switch provider (Table 3) is evident from the majority that rated the NHIS highly, would renew their membership card and recommend the NHIS to others (Table 5). These findings may however be interpreted with some caution since contextual factors in Ghana may not compare favourably with those in Hall’s study setting, which was conducted among members of a Health Maintenance Organization (HMO) in North Carolina-USA. Many subscribers in Ghana may not have the financial ability to opt out of the NHIS for private insurance as the case could be in high-income countries; and may, therefore, have to accept what is available to them despite challenges that they may be faced with. It is also worth noting that with only a fifth of respondents rating capitation payment, one may wonder whether the poor response to the rating of capitation payment could not mean a silent protest against it, considering that many Ghanaians have a culture of not willing to openly speak to issues about which they have reservations or those with which they are not comfortable.

### Subscriber perception about the positive attributes of, and attitude towards capitation payment

One interesting thing that may be of interest to stakeholders in the debate around the capitation payment policy in Ghana is the seeming conflict between subscriber perception about the positive attributes of capitation on the one hand, which is generally affirmative as expressed in the results presented in Table 4; and, on the other hand, respondents’ attitude towards the capitation payment policy which point to the negative as may be deduced from the results presented in Table 5. Attributes such as capitation potential to drive down cost, improve quality of care, including better diagnosis and treatment, are perceived positively, while many respondents showed agreement with statements such as that capitation is politically motivated and causes frustration at the health facilities (Table 5). This gives the impression that the communication messages on the positive attributes of capitation payment may have gone down well with the public and that their negative attitude towards the policy may have been influenced by the negative media reportage on the policy and the frustrations subscribers may have experienced at the health facilities at the initial stages of the introduction of the payment policy. A more qualitative study to explore and understand this phenomenon would be required.

Some limitations may identify with the study. The first is the disproportionate balance in representation between male and female respondents. It is, however, common in Ghana for more females than males to access health care services and that their opinion about the capitation payment could provide a reasonable reflection of the situation. A second limitation may be the relatively low sample size which was used for the chi-square statistical analysis, a result from data cleaning and excluding respondents without care provider and lack of knowledge on capitation. That notwithstanding, results from the analysis provides a reasonable approximation of the perception of insured clients about the capitation payment policy in the Ashanti region of Ghana. The study may also be limited by courtesy bias. This is because, although respondents expressed misgivings about the capitation payment policy, they expressed high trust in the NHIS and in their primary care providers in providing quality care for them. This is however not surprising because of the traditional respect patients, and Ghanaians in general, have for their care providers.

## Conclusion

We conclude that respondents have a high trust in their primary care provider giving them quality care under capitation payment despite their negative attitude towards capitation payment. This finding provides useful reflection of subscriber side of the issue to the public debate that characterized the introduction of capitation payment in Ashanti. Hitherto, the debate had been dominated by politicians, providers and civil society groups leaving out the subscribers who had no access to the radio and television to express their opinion on the subject matter.

We also conclude that health insurance subscribers are guided by proximity and quality of care considerations in their choice of provider. This provides useful insight for policy-makers in their determination to enforce quality of care standards in the health delivery system. Whereas findings, especially those relating to subscriber trust in their primary care provider provide potential support for the scaling up of capitation payment in Ghana, the NHIA would, none the less, have to address itself to the negative perceptions of subscribers about the capitation payment policy. This would require the NHIA to invest in IEC to sensitize the public on the positive effects of capitation payment and to address the negative perception about it.

## Abbreviations

CHPS, Community-based Health Planning Service; DRG, Diagnosis-Related Grouping; EA, Enumeration Area; EPI, Expanded Programme on Immunization; FFS, Fee-for-service; GLSS, Ghana Living Standard Survey; GSS, Ghana Statistical Service; HMO, Health Maintenance Organization; IEC, Information, Education and Communication; NHIA, National Health Insurance Authority; NHIS, National Health Insurance Scheme; PES, Post-Enumeration Survey; PPP, Preferred Primary Care Provider; WHO, World Health Organization

## Additional file

Additional file 1: Household interview questionnaires. (DOCX 227 kb)
