# Supplementary material for: Does capitation payment under national health insurance affect subscribers’ trust in their primary care provider? a cross-sectional survey of insurance subscribers in Ghana
Source: BMC Health Serv Res. 2016 Aug 24;16(1):437. doi: 10.1186/s12913-016-1622-0 (PMC4997684; doi:10.1186/s12913-016-1622-0)
Supplement: Additional file 1: — Household interview questionnaires. (DOCX 227 kb) [file 12913_2016_1622_MOESM1_ESM.docx]

# PART TWO

**SUBSCRIBER (HOUSEHOLD) INTERVIEWS**

| Title of Study: | Provider payment reforms within the National Health Insurance Scheme:  Monitoring and Evaluation of Capitation as a provider payment mechanism for primary out-patients services. |
| --- | --- |
| Principal Investigator: | Francis-Xavier Andoh-Adjei |
| Certified Protocol Number | UG-ECH 057/13-14 |

Section B– CONSENT TO PARTICIPATE IN RESEARCH

**General Information about Research**

We are conducting a study on the implementation of capitation as a provider payment mechanism in order to assess its effect on health service delivery at the primary out-patient department of NHIS-credentialed facilities and to explore NHIS card bearers’ perception of quality of service and how that perception influence their insurance membership status and health seeking behavior. We shall need about one hour of your time to respond to some questions that we will ask you for answers.

**Benefits/Risk of the study**

The results of the study will help the National Health Insurance Authority to improve its operations for the benefit of all stakeholders and the nation as a whole.

**Confidentiality**

We shall not disclose the identity of any person who responds to our questions or who provide us with any information that will help us in this research. Names of persons who respond to our questions will not be written in any of our reports or articles that will be published for international consumption.

Apart from the university authorities and the research team, no other persons will have access to the research records. The final results, which will be in the form of a report, will however be shared with all stakeholders, that is the NHIA, providers and subscribers at NHIS stakeholders’ forum. Articles will also be prepared from the reports and published in international peer-review journals.

**Compensation**

This is an academic study intended to help NHIA improve on its operations for the benefit of the entire society and therefore there will not be any incentive whatsoever for people who volunteer to respond to the questions that we shall ask.

**Withdrawal from Study**

Your participation in this study is voluntary and you are free to withdraw from the study or even refuse to answer any question if you wish and nobody will fault you for your decision to withdraw from the study or your refusal to answer any question.

**Contact for Additional Information**

In case you have any questions or information about this research that you may want to convey to us, you may direct them to the following: **Francis-Xavier Andoh-Adjei, Head of Planning, Monitoring & Evaluation and International Relations, National Health Insurance Authority, 36-6th Avenue, Ridge, PMB, Ministries Post Office, Accra. You can also reach him on telephone numbers: 024 461 3747 or 020 923 4905.**

Section C- VOLUNTEER AGREEMENT

**"I have read or have had someone read all of the above, asked questions, received answers regarding participation in this study, and am willing to give consent for me, my child/ward to participate in this study. I will not have waived any of my rights by signing this consent form. Upon signing this consent form, I will receive a copy for my personal records."**

Name of Volunteer:

Signature or mark of volunteer Date

**If volunteers cannot read the form themselves, a witness must sign here:**

I was present while the benefits, risks and procedures were read to the volunteer. All questions were answered and the volunteer has agreed to take part in the research.

Name of witness

Signature of witness Date

I certify that the nature and purpose, the potential benefits, and possible risks associated with participating in this research have been explained to the above individual.

Name of Research Assistant

Signature of Research Assistant Date

INDENTIFICATION

| NAME OF RESPONDENT: ................................................................................. | |  |
| --- | --- | --- |
| GENDER: (MALE=1, FEMALE=2) | |  |
| TELEPHONE NUMBER: .................................................................................... | |  |
| REGION: (ASHANTI=1, VOLTA=2, CENTRAL=3) | |  |
| DISTRICT: ................................................................................................. ............ | |  |
| ENUMERATION AREA BASE NAME ………………………………………… | |  |
| URBAN/RURAL (URBAN=1, RURAL=2) | |  |
| COMMUNITY (CITY=1, LARGE TOWN=2, SMALL TOWN=3 VILLAGE=4) | |  |
|  | |  |
| LANGUAGE OF QUESTIONNAIRES: **ENGLISH**  LANGUAGE OF INTERVIEW LANGUAGE OF RESPONDENT  WAS TRANSLATOR USED? (YES=1, NO=2)  ***LANGUAGE CODES: (ENGLISH=1, AKAN=2, GA=3, EWE=4, NZEMA=5,  DAGBANI=6, OTHER=7).........................................  SPECIFY | |  |
|  | |  |
| **NAME OF RESEARCH ASSISTANT** | **SIGNATURE** | **DATE** |
|  |  | DAY  MONTH RESULTS |
| NAME OF SUPERVISOR | SIGNATURE | DAY  MONTH  RESULTS |
| RESULTS CODES: COMPLETED=1, PARTIALLY COMPLETED=2, REFUSED3, OTHER=4 …………………  (SPECIFY) | | |

#### SECTION 1: RESPONDENT’S BASIC SOCIO-DEMOGRAPHICS

| **I would like to ask some questions about yourself and I shall be grateful for your answers.** | | | |
| --- | --- | --- | --- |
| **NO.** | **QUESTIONS & FILTERS** | **CODING CATEGORIES** | |
| 1. | How old were you at your last birthday? | AGE IN COMPLETE YEARS |  |
| 2. | What is your marital status? | 1. Never married 2. Married 3. Separated 4. Divorced 5. Widowed 6. Cohabitating |  |
| 3. | What is the highest level of school you attended? | 1. Primary 2. Middle/JSS 3. Secondary/SSS 4. Higher 5. Never attended school |  |
| 4. | What is your employment status? | 1. Government employee 2. Non-government employee 3. Self-employed 4. Non-paid (volunteer)   5. Student   1. Home-maker 2. Retiree   8. Unemployed/(able to work) |  |
| 5. | During the last 12 months, what has been your main occupation?  **NOTE:**  **Main occupation refers to work on which respondent spent most of his/her time or earned him/her the most income during the past year.** | 1. Senior official/manager 2. Professional (engineer, doctor, teacher, etc) 3. Technician 4. Service/sales worker 5. Agric. /fishery worker 6. Plant/machine operator/assembler 7. Elementary worker (street Food vendor, etc) 8. Other [specify]: ………….. |  |
| 6. | How long have you been a registered member of the NHIS? | 1. <1 year 2. 1-2 years 3. >2-3 years 4. >3-5 years 5. > 5 years |  |

#### SECTION 2: SUBSCRIBER HEALTH CARE UTILIZATION

| **Now, I would like to ask you about your health care utilization.** | |
| --- | --- |
| 7. How many times did you go for treatment during the past year (**2013**)? | 1. ONE TIME 2. TWO TIMES 3. THREE TIMES 4. MORE THAN 3 TIMES 5. NONE AT ALL |
| 8. When was the last time that you needed health care and you went to a health facility for treatment? | 1. 1-3 MONTHS AGO 2. 4-6 MONTHS AGO 3. 7-9 MONTHS AGO 4. 10-12 MONTHS AGO 5. MORE THAN 12 MONTHS AGO |
| 9. Which type of health facility did you go for treatment the last time? | 1. CHPS COMPOUND 2. MATERNITY HOME 3. HEALTH CENTER 4. CLINIC 5. HOSPITAL |
| 10. The facility you visited the last time is operated by | 1. GOVERNMENT 2. PRIVATE 3. MISSION 4. OTHER (SPECIFY)    - 1. ……………………………. |
| 11. Is the facility you visited last time the place where you always go for treatment? | 1. Yes 2. NO |
| 12. Which of the following health care personnel attended to you? | 1. MEDICAL DOCTOR (including any specialist) 2. MEDICAL ASSISTANT 3. NURSE 4. MIDWIFE 5. COMMUNITY HEALTH NURSE 6. HEALTH ASSISTANT 7. OTHER (SPECIFY)………………………….... |
| 13. How long did it take for you to be seen by the prescriber? | 1. < 15 MINS 2. 15-30 MINS 3. 31-45 MINS 4. 46-60 MINS 5. > 60 MINS |
| 14. How much time did the Prescriber spend with you in the consulting room? | 1. < 5 MINS 2. 5-10 MINS 3. 10-15 MINS 4. > 15 MINS |
| 15. What ailment did you take to the facility for treatment during your last visit? | Name of ailment: |

| 16. Were you referred to the laboratory for investigation during your last visit? | 1. YES 2. NO **(If “NO” , go to 17)** |
| --- | --- |
| 17. Did you do all or some of the laboratory test at the facility? | 1. YES, ALL 2. YES, SOME 3. NO, NONE AT ALL |
| 18. Have you ever been referred to any private laboratory for tests that you think could have been done in the facility? | 1. YES 2. NO |
| 19. Did the Doctor/Nurse prescribe medicines for you? | 1. YES 2. NO **(If “NO” go to 22)** |
| 20. Did you receive all the medicines prescribed for you in the facility? | 1. YES, ALL 2. YES, SOME 3. NO, NOT AT ALL |
| 21. How would you rate the medicines that were prescribed and given to you? | 1. VERY GOOD 2. GOOD 3. BAD 4. VERY BAD |
| 22. Did you have to pay any money at the facility? | 1. YES 2. NO **(If “NO”, go to 24)** |
| 23. How much money did you pay at last visit for any of the following, if applicable? | 1. LABORATORY: GH¢………….………… 2. MEDICINES : GH¢……………………….. 3. OTHER : GH¢ ………………………. |
| **Total** | **GH¢……………………….** |
| 24. Have you ever been referred to another (level care) facility for treatment that you think could have been handled at this facility? | 1. YES 2. NO |
| 25. In the last 12 months that you have had experience with the provider, how would you rate your satisfaction with services provided to you? | 1. VERY GOOD 2. GOOD 3. BAD 4. VERY BAD |

#### SECTION 3: SUBSCRIBER PERCEPTION OF QUALITY OF CARE-GENERAL

Now, I would like to find out your opinion about care provision in the facility where you go for treatment (**in reference to your last visit to the facility**) and I shall be grateful for your responses. The opinions are expressed in statements and your response will be to strongly agree, agree, disagree or strongly disagree with the statement in the following rankings:

#### Strongly agree (4) Agree (3) Dis-agree (2) Strongly dis-agree (1) Don’t know (8)

|  | **Staff availability & prompt attention** | |  |
| --- | --- | --- | --- |
| 26. | There was a prescriber available to attend to me. | \| **4** \| **3** \| **2** \| **1** \| **8** \| \| --- \| --- \| --- \| --- \| --- \| |  |
| 27. | I was able to see the prescriber within 30  mins. | \| **4** \| **3** \| **2** \| **1** \| **8** \| \| --- \| --- \| --- \| --- \| --- \| |  |
|  | **Dignity and respect** | |  |
| 28. | The Nurses were courteous towards me. | \| **4** \| **3** \| **2** \| **1** \| **8** \| \| --- \| --- \| --- \| --- \| --- \| |  |
| 29. | The Nurses treated me with dignity. | \| **4** \| **3** \| **2** \| **1** \| **8** \| \| --- \| --- \| --- \| --- \| --- \| |  |
|  | **Confidentiality** | |  |
| 30. | The consulting room was such that when I was telling my condition to the Doctor / Nurse, no one else could hear me. | \| **4** \| **3** \| **2** \| **1** \| **8** \| \| --- \| --- \| --- \| --- \| --- \| |  |
| 31. | The nurses keep clients’ health information secret and confidential. | \| **4** \| **3** \| **2** \| **1** \| **8** \| \| --- \| --- \| --- \| --- \| --- \| |  |
|  | **Service quality** | |  |
| 32. | The prescriber made a good diagnosis when I went for treatment the last time. | \| **4** \| **3** \| **2** \| **1** \| **8** \| \| --- \| --- \| --- \| --- \| --- \| |  |
| 33. | The treatment I got from the prescriber was effective for recovery and cure. | \| **4** \| **3** \| **2** \| **1** \| **8** \| \| --- \| --- \| --- \| --- \| --- \| |  |
| 34. | The medicines that were prescribed for me were very good for the ailment. | \| **4** \| **3** \| **2** \| **1** \| **8** \| \| --- \| --- \| --- \| --- \| --- \| |  |

|  | **Communication** | |  |
| --- | --- | --- | --- |
| 35. | The prescriber made time to discuss my health condition and the required treatment with me. | \| **4** \| **3** \| **2** \| **1** \| **8** \| \| --- \| --- \| --- \| --- \| --- \| |  |
| 36. | He/She explained everything about the treatment to me before I left. | \| **4** \| **3** \| **2** \| **1** \| **8** \| \| --- \| --- \| --- \| --- \| --- \| |  |
| 37. | He/she advised me on the side effects of treatment that he prescribed for me. | \| **4** \| **3** \| **2** \| **1** \| **8** \| \| --- \| --- \| --- \| --- \| --- \| |  |
| 38. | He/She spent time to advise me on preventive care. | \| **4** \| **3** \| **2** \| **1** \| **8** \| \| --- \| --- \| --- \| --- \| --- \| |  |
| 39. | He/She opened up to me for questions about the treatment s/he gave. | \| **4** \| **3** \| **2** \| **1** \| **8** \| \| --- \| --- \| --- \| --- \| --- \| |  |
|  | **Autonomy** | |  |
| 40. | The prescriber gave me the option to accept or to refuse the treatment s/ he prescribed for me. | \| **4** \| **3** \| **2** \| **1** \| **8** \| \| --- \| --- \| --- \| --- \| --- \| |  |
|  | **Accommodation/cleanliness** | |  |
| 41. | The last time I visited the facility, the seats at the waiting area were enough to seat everybody who came for treatment. | \| **4** \| **3** \| **2** \| **1** \| **8** \| \| --- \| --- \| --- \| --- \| --- \| |  |
| 42. | During my last visit to the facility, the environment, including the toilet facilities, was neat. | \| **4** \| **3** \| **2** \| **1** \| **8** \| \| --- \| --- \| --- \| --- \| --- \| |  |
| 43. | During my last visit, there was no congestion at the health facility. | \| **4** \| **3** \| **2** \| **1** \| **8** \| \| --- \| --- \| --- \| --- \| --- \| |  |
| 44. | During my last visit to the facility, the waiting area was well ventilated. | \| **4** \| **3** \| **2** \| **1** \| **8** \| \| --- \| --- \| --- \| --- \| --- \| |  |

**SECTION 4: KNOWLEDGE AND PERCEPTION ABOUT CAPITATION**

| Now, I would like to find out your knowledge about capitation and why you chose a particular provider as your preferred primary care provider (PPP). | | |
| --- | --- | --- |
| **Question** | **Response** | **Response**  **code** |
| 45. Do you know about capitation? | 1. YES  **2. NO [If “NO” go to 45]** |  |
| 46. How did you get to know of capitation? | 1. SCHEME STAFF 2. PROVIDER 3. RADIO 4. TELEVISION 5. COLLEAGUE 6. OTHER (SPECIFY) ……………………………. |  |
| 47. Which of the following statements expresses your understanding of capitation? | 1. ADVANCE PAYMENT TO PROVIDERS FOR ALL OPD SERVICES 2. ADVANCE PAYMENT TO PROVIDERS FOR SOME OPD SERVICES 3. ADVANCE PAYMENT TO PROVIDERS FOR BOTH OPD AND IPD SERVICES   4. DON’T KNOW |  |
| **Capitation and choice of PPP (Not applicable to Volta and Central)** | | |
| 48. Did you understand the concept of capitation before it was implemented? | 1. YES  2. NO |  |
| 49. Do you have a Preferred Primary  Care Provider (PPP)? | 1. YES 2. NO |  |
| 50. Which one of the following is your Preferred Primary Provider (PPP)? | 1. CHPS COMPOUND 2. MATERNITY HOME 3. HEALTH CENTER (GOVERNMENT) 4. HEALTH CENTER (PRIVATE/MISSION) 5. CLINIC (GOVERNMENT) 6. CLINIC PRIVATE/MISSION) 7. HOSPITAL (GOVERNMENT) 8. HOSPITAL (PRIVATE/MISSION)   9. OTHER (SPECIFY)  ………………………………. |  |

| 51. Did you choose the PPP yourself? | | | 1. YES 2. NO, ASSIGNED BY SCHEME 3. NO, ASSIGNED ON ATTENDANCE AT   FACILITY **(If 2 or 3, go to 50 )** | | |  | | |
| --- | --- | --- | --- | --- | --- | --- | --- | --- |
| 52. What is your main reason for choosing that provider as your (PPP)? | | | 1. CLOSENESS TO ME 2. PROVIDE GOOD TREATMENT 3. GOOD ATTITUDE OF STAFF 4. NO OTHER ALTERNATIVE 5. OTHER (SPECIFY)   ….…………………………………. | | |  | | |
| 53. What do you understand by choosing a PPP? | | | 1. CHOOSE 3 AND ATTEND ANY OF THEM   WHEN SICK.   1. CHOOSE 3 BUT ATTEND ONLY ONE WHEN SICK. 2. CHOOSE 3 AND ATTEND ANYONE DEPENDING ON THE HEALTH CONDITION. 3. DON’T UNDERSTAND IT. | | |  | | |
| 54. How long do you think one should stay with his/her PPP before (s) he changes if (s) he wants to? | | | 1. 3 MONTHS 2. 6 MONTHS 3. 9 MONTHS 4. 12 MONTHS 5. ANYTIME ONE WANTS TO CHANGE 6. DON’T KNOW | | |  | | |
| Now, I would like to find out your experience of, and opinion about your preferred primary care provider on aspects of their services and I shall be grateful for your responses. These are expressed in statements and your response will be to strongly agree, agree, disagree or strongly dis-agree with the statement in the follow rankings**: (NOT APPLICABLE TO VOLTA AND CENTRAL)**  **Strongly agree (4) Agree (3) Dis-agree (3) Strongly dis-agree (2) Don’t know (8)** | | | | | | | | |
|  | **Perceived reason(s) behind capitation** | | | | | |  | |
| 55. | Capitation is meant to drive down cost of health care to a reasonable level. | | | \| **4** \| **3** \| **2** \| **1** \| **8** \| \| --- \| --- \| --- \| --- \| --- \| | | |  | |
| 56. | Capitation is meant to improve the quality of care. | | | \| **4** \| **3** \| **2** \| **1** \| **8** \| \| --- \| --- \| --- \| --- \| --- \| | | |  | |
| 57. | Capitation is meant to punish the people in  Ashanti region. | | | \| **4** \| **3** \| **2** \| **1** \| **8** \| \| --- \| --- \| --- \| --- \| --- \| | | |  | |
| 58. | Capitation was brought to Ashanti because of politics. | | | \| **4** \| **3** \| **2** \| **1** \| **8** \| \| --- \| --- \| --- \| --- \| --- \| | | |  | |
|  |  | | |  | | |  | |
|  | **Responsiveness under capitation** | | | | | |  | |
| 59. | | If one has not enrolled with any provider and  (s)he goes for treatment, they enroll him/her on the spot and provide the treatment needed | | | \| **4** \| **3** \| **2** \| **1** \| **8** \| \| --- \| --- \| --- \| --- \| --- \| | | |  |
| 60. | | If one has not enrolled with any provider and he goes for treatment, they ask him/her to pay for the cost of treatment. | | | \| **4** \| **3** \| **2** \| **1** \| **8** \| \| --- \| --- \| --- \| --- \| --- \| | | |  |
| 61. | | If one is on capitation, (s) he pays extra money for the treatment (s) he gets. | | | \| **4** \| **3** \| **2** \| **1** \| **8** \| \| --- \| --- \| --- \| --- \| --- \| | | |  |
|  | | **Perceived effects of capitation** | | | | | |  |
| 62. | | Capitation is contributing to the death of people in Ashanti region. | | | \| **4** \| **3** \| **2** \| **1** \| **8** \| \| --- \| --- \| --- \| --- \| --- \| | | |  |
| 63. | | Capitation is causing frustration to the insured at the health facilities. | | | \| **4** \| **3** \| **2** \| **1** \| **8** \| \| --- \| --- \| --- \| --- \| --- \| | | |  |
| 64. | | Since Capitation started, providers to refer potential primary care cases to higher levels of care. | | | \| **4** \| **3** \| **2** \| **1** \| **8** \| \| --- \| --- \| --- \| --- \| --- \| | | |  |
| 65. | | Capitation is a good way of stopping NHIS members from moving from one provider to another without any good reason. | | | \| **4** \| **3** \| **2** \| **1** \| **8** \| \| --- \| --- \| --- \| --- \| --- \| | | |  |
|  | | **Continuity of care** | | | | | |  |
| 66. | | Capitation is good because I have only one provider who knows my health problems for better diagnosis and treatment. | | | \| **4** \| **3** \| **2** \| **1** \| **8** \| \| --- \| --- \| --- \| --- \| --- \| | | |  |
| 67. | | Capitation is good because if my PPP cannot take care of my disease, s/he will refer me to another place for care. | | | \| **4** \| **3** \| **2** \| **1** \| **8** \| \| --- \| --- \| --- \| --- \| --- \| | | |  |
| 68. | | Capitation is good because, now you know who your primary care provider is, and (s)he also knows you so treatment becomes very easy and effective. | | | \| **4** \| **3** \| **2** \| **1** \| **8** \| \| --- \| --- \| --- \| --- \| --- \| | | |  |
|  | |  | | |  | | |  |
|  | | Now, I would like you to share with me your final impressions about capitation and the NHIS | | | | | |  |
| 69. | | How will you rate the trust that your primary care provider will give you the best of treatment that you expect? | | | 1. VERY HIGH 2. HIGH 3. LOW 4. VERY LOW | | |  |
| 70. | | Do you intend changing your PPP after the 6 months period? | | | 1. YES 2. NO **(If “NO” go to 70)** | | |  |
| 71. | | Why would you want to change your PPP? | | | 1. POOR SERVICE QUALITY 2. NO SKILLED STAFF AVAILABLE 3. NO GOOD MEDICINES 4. OTHER: | | |  |
| 72. | | Considering your experience with capitation, would you renew your card when it expires? | | | 1.YES  2. NO | | |  |
| 73. | | Considering your experience with capitation, would you recommend anyone without insurance card to register with the NHIS? | | | 1.YES  2. NO | | |  |
| 74. | | On the basis of your experience so far, how would you rate the Capitation? | | | 1. VERY GOOD 2. GOOD 3. BAD 4. VERY BAD | | |  |
| 75. | | On the basis of your experience with the  NHIS so far, how would you rate Scheme? | | | 1. VERY GOOD 2. GOOD 3. BAD 4. VERY BAD | | |  |

Thank you very much for your cooperation. If there are any questions that you want to ask me, or any issues that you want me to explain or clarify to you, I shall be very happy to do so.
